# Supplementary material for: Integrative machine learning approach to risk prediction for dementia and Alzheimer’s disease
Source: GeroScience. 2025 Aug 27;48(2):3007–23. doi: 10.1007/s11357-025-01828-x (PMC12972432; doi:10.1007/s11357-025-01828-x)
Supplement: Supplementary file 1 — Supplementary file1 (DOCX 4951 KB) [file 11357_2025_1828_MOESM1_ESM.docx]

**Integrative Machine Learning Approach to Risk Prediction for Dementia and Alzheimer’s Disease**

# Amos Stern^1^, Michal Linial^2, *^

^1^The Rachel and Selim Benin School of Computer Science and Engineering, The Hebrew University of Jerusalem, Jerusalem, Israel

^2^Department of Biological Chemistry, The Life Science Institute, The Hebrew University of Jerusalem, Israel

Supplementary **Text** **S1**

**Table S1:** Statistical test of age distributions differences between AD and controls before and after the age-maching protocol

| **AD vs. Control (original)** | | |
| --- | --- | --- |
| **Test** | **statistic** | **p-value** |
| KS-test | 0.481 | 0 |
| Mann–Whitney U | 1078213763 | 0 |
| T-test | 53.297 | 0 |

| **AD vs. Control (age-matched)** | | |
| --- | --- | --- |
| **Test** | **statistic** | **p-value** |
| KS-test | 9.05E-05 | 1 |
| Mann–Whitney U | 104106113.5 | 0.992 |
| T-test | -0.017 | 0.985 |

**Table S2.** Model performance summary with OT genes and PWAS scoring

| **Model (Abbreviated name)** | **AUC (mean)** | **AUC (std)** |
| --- | --- | --- |
| female_all | 0.773 | 0.012 |
| female_all_PWAS_OT-genetics | 0.769 | 0.014 |
| female_all_PWAS_OT-global | 0.767 | 0.013 |
| all | 0.761 | 0.008 |
| all_PWAS_OT-global | 0.760 | 0.008 |
| all_PWAS_OT-genetics | 0.757 | 0.008 |
| male_all_PWAS_OT-genetics | 0.723 | 0.015 |
| male_all_PWAS_OT-global | 0.722 | 0.018 |
| male_all | 0.722 | 0.012 |
| only_PWAS_OT-genetics | 0.693 | 0.009 |
| only_PWAS_OT-global | 0.692 | 0.009 |

**Table S3.** Feature importance for the top features of the “all” model summary results of 10 iterations.

| **Feature name** | **Mean**  **importance** | **Std**  **importance** | **Mean**  **rank** | **Std**  **rank** |
| --- | --- | --- | --- | --- |
| rs429358 | 0.4693 | 0.0224 | 1 | 0.0000 |
| Filtered_ICD | 0.1901 | 0.0335 | 2 | 0.0000 |
| AgeLastEpisodeOfDepression | 0.1059 | 0.0285 | 3.5 | 0.9718 |
| AgeOfStopSmoking | 0.0800 | 0.0080 | 3.9 | 0.5676 |
| Medication | 0.0593 | 0.0090 | 5.3 | 0.6749 |
| rs7412 | 0.0535 | 0.0150 | 5.4 | 1.0750 |
| IllnessOfFather | 0.0204 | 0.0112 | 8.3 | 1.0593 |
| Qualifications | 0.0233 | 0.0138 | 9.6 | 3.4705 |
| IllnessOfMother | 0.0144 | 0.0078 | 10.5 | 1.8409 |
| DentalProblems | 0.0158 | 0.0104 | 11.5 | 5.6224 |
| IllnessInjuriesStressLast2Years | 0.0095 | 0.0093 | 14 | 5.1208 |
| BMI | 0.0109 | 0.0082 | 14.65 | 10.1764 |
| VigorousPhysicalActivity_NumDaysWeek | 0.0069 | 0.0052 | 17.2 | 10.9929 |
| LifeQuality | 0.0082 | 0.0081 | 19.2 | 13.2669 |

Supplementary **Fig. S1**


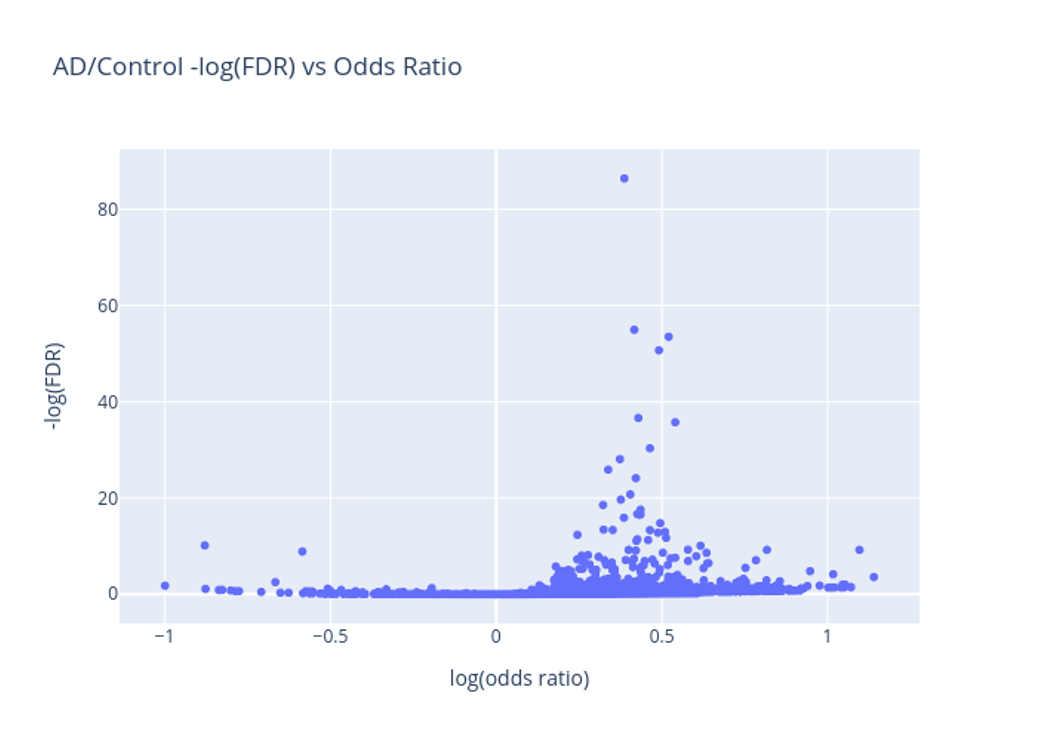


**Figure S1.** Plot of -log10(q-value FDR) vs. log2(odds ratio; OR) of Fisher's exact test for ICD-10 diagnosis of AD and control groups. The figure captures the effect size and statistical significance.

Supplementary **Fig. S2**


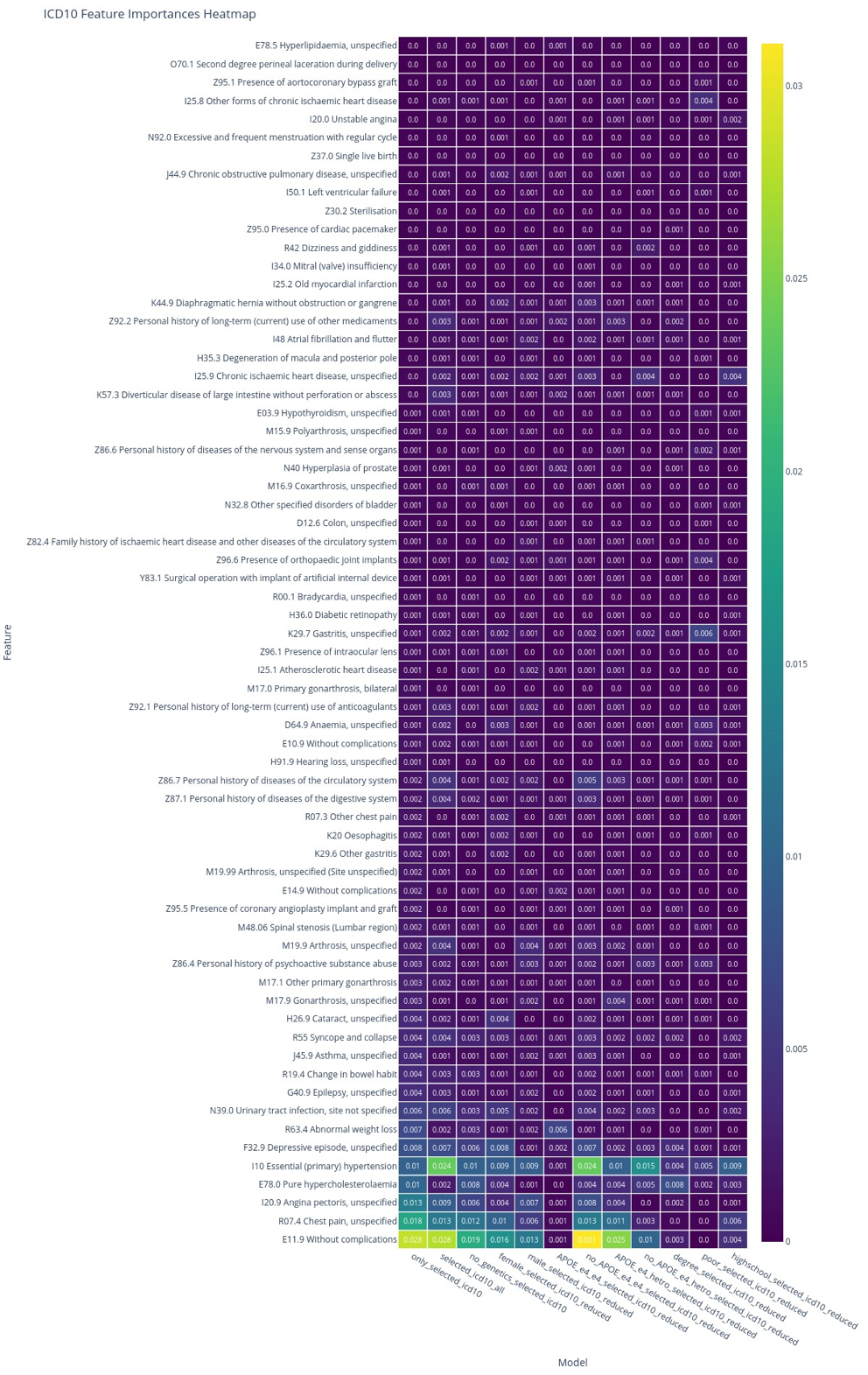
**Fig. S2.** Heatmap of mean SHAP importances of all 66 ICD-10 features used in model training. ICD-10 features are sorted by the only_selected_icd10 model mean feature importance. Importance values are rounded with 2 digit precision.
